# High-resolution real-time mechanochromic tactile sensors

**DOI:** 10.1126/sciadv.aee5236

**Published:** 2026-07-03

**Authors:** Giacomo Sasso, Alessandro Pagani, Aaron M. Duncan, Gianni Pedrizzetti, Nicola Pugno, James J. C. Busfield, Federico Carpi

**Affiliations:** ^1^ School of Engineering and Materials Science, Queen Mary University of London, London, UK.; ^2^ Department of Industrial Engineering, University of Florence, Florence, Italy.; ^3^ Department of Engineering and Architecture, University of Trieste, Trieste, Italy.; ^4^ Mechano-X Labs, Department of Civil, Environmental and Mechanical Engineering, University of Trento, Trento, Italy.

## Abstract

High-resolution, real-time tactile sensing is essential for robotic tasks that demand accurate, dynamic detection of contact morphology and pressure distribution, such as grasping and manipulation of delicate, slippery, or irregularly shaped objects. Existing technologies, however, face a fundamental trade-off between spatial resolution and response speed. Taxel-based sensors (e.g., capacitive, resistive, or piezoelectric) operate in real time but are intrinsically limited in resolution by taxel size, spacing, wiring, and cross-talk; even deep learning–based tactile super-resolutions rarely surpass ∼1 millimeter. Finer resolutions can be achieved with vision-based tactile sensors using just a camera, although the computation required to transform raw images into three-dimensional contact maps inherently introduces latency. Here, we present mechanochromic tactile sensors that directly encode mechanical strain into spatially resolved structural colors, enabling vision-based tactile sensing with an unprecedented combination of high resolution, real-time operation, and intrinsic simplicity. The devices consist of a stretchable mechanochromic Bragg reflector embedded between two soft silicone layers, whose thickness can be tailored to precisely map contact pressure or strain. As an example, we present topological maps of a fingertip, a one-penny coin, and a leaf, with ∼100 micrometer resolution. In comparison to the most performing vision-based tactile sensors, this was achieved without requiring any deep learning–based data enhancement and without introducing any computational latency. The straightforward applicability of this mechanochromic strategy to enhance vision-based tactile sensing in a simple yet powerful way underscores its transformative potential for uses as diverse as robotic gripping and handling, tactile product inspection, and enhanced human-robot interaction.

## Teaser

Mechanochromic sensors allow for vision-based tactile sensing in real time at high spatial resolutions.

## INTRODUCTION

Tactile sensing plays an ever-growing role in various robotic systems interacting with the physical world, such as anthropomorphic hands and grippers. Such machines require soft/flexible surfaces capable of spatially distributed tactile sensing and conformability to objects of variable geometrical complexity (*1*–*3*). In particular, the ability to build spatial maps of contact morphology and pressure at high resolution and in real time is fundamental for high-level robotic functions as diverse as object detection, grasping and manipulation, environmental tactile mapping, and enhanced human-machine interaction (*4*–*7*).

Existing tactile sensing strategies typically rely on piezocapacitive (*8*–*10*), piezoresistive (*11*–*13*), piezoelectric (*14*, *15*), triboelectric (*16*, *17*), or magnetic effects (*18*, *19*). These technologies transduce mechanical deformations into electrical information through arrays of sensing units (taxels). Usually, they do not require time-consuming processing, enabling real-time operation. However, when such arrays are embedded into soft/flexible tactile interfaces, their spatial resolution is limited by the taxel size, spacing, and cross-talk, along with the surface area required to route stretchable/flexible interconnects to peripheral electronics (*20*). Recently, combinations of taxel arrays with deep learning–based strategies have been used to achieve tactile super-resolutions (i.e., spatial resolutions finer than the taxel-constrained physical limit), although they remain confined to the order of 1 mm (*1*).

To overcome the limitations of taxel-based technologies, various approaches have been proposed. Xu *et al.* (*21*), for example, replaced conventional arrays of small elastomeric capacitors with a layered structure, comprising two capacitive membranes, virtually segmented into multiple sensing zones through a multifrequency interrogation method. That strategy enabled spatially resolved sensing without the need for physically segmented taxels (*21*). However, achieving high-resolution mapping for fine tactile sensing with such a method remains challenging. Alternatively, electrical impedance tomography (EIT) techniques can be used for contact localization from electrical measurements taken exclusively from the periphery of conductive surfaces (*22*–*24*). While promising in terms of wiring simplification, current EIT-based systems remain limited to spatial resolutions on the order of 10 mm (*23*).

**Fig. 1.**
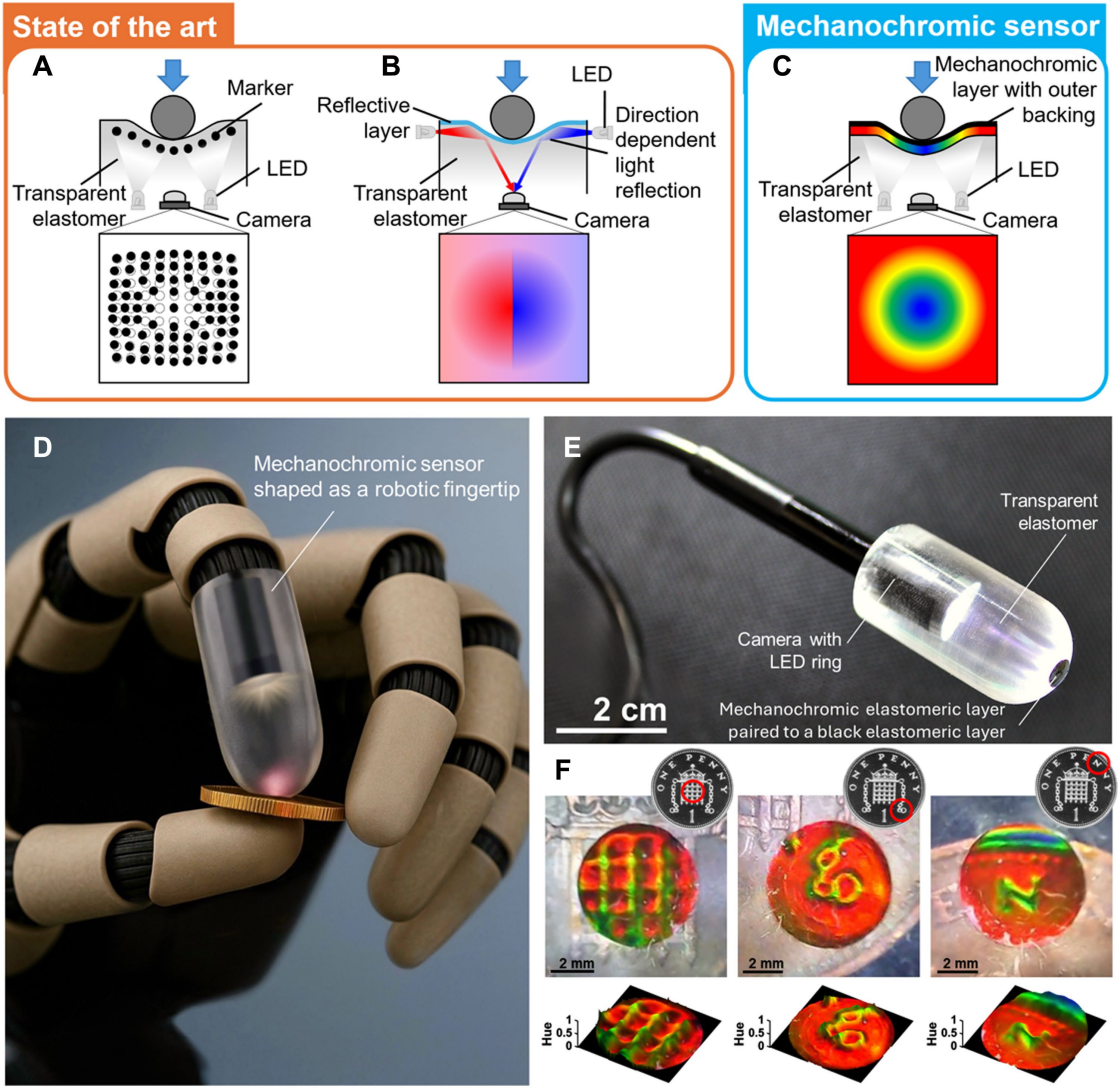
Mechanochromic tactile sensing: Comparison with state-of-the-art vision-based sensing strategies and example of embodiment in a robotic finger. (**A** to **C**) Schematic diagrams of the working principles of the two most used conventional approaches for vision-based tactile sensing (marker displacement and directional light reflection), in comparison with the mechanochromic strategy proposed in this work. (**D**) Conceptual illustration of an example of an embodiment of the mechanochromic sensor, integrated into the structure of an anthropomorphic robotic finger. (**E**) Photograph of a prototype implementation of the sensorized robotic fingertip, where a transparent elastomeric cap was coated with a small circular patch (5 mm in diameter) of the mechanochromic bilayer. (**F**) Examples of images taken by the robotic fingertip’s internal camera when the mechanochromic interface contacted different parts of a one-penny coin (top panels), and corresponding 3D hue maps (bottom panels). A video of the real-time tactile sensing tests is available as movie S1.

An effective alternative is offered by vision-based (or optical) tactile sensing (*25*, *26*). It replaces an array of taxels with just a camera, which captures contact-induced deformations of a soft elastomeric interface. The camera is usually positioned to monitor the tactile interface from the side opposite to where contact occurs, i.e., from the inside of an enclosure created behind it. Tactile events are typically detected using two main strategies: (i) tracking the displacement of embedded visual markers (*25*, *27*–*30*), such as in TacTip sensors (*31*), as illustrated in Fig. 1A; (ii) measuring changes in reflection patterns of distinct lights, typically red-green-blue or white, internally projected from different directions (*25*, *32*–*35*), such as in GelSight sensors (*36*, *37*), as shown in Fig. 1B.

The second category falls within the broader class of intensity-based methods, which can use either reflection- or transmission-based strategies, although the reflection mode is the most investigated (*25*, *26*). Moreover, hybrid approaches that combine both marker-based and intensity-based methods have been proposed to leverage complementary advantages (*25*, *38*–*40*). Marker-based sensing enables the detection of contact pressures and surface textures, but it is inherently constrained in spatial resolution by the markers’ density, i.e., their size and spacing. On the other hand, improved sensing performance can be achieved with directional light reflection-based sensing; by using accurate configurations of illumination and deep learning–based data-driven image processing, the resolution can be in the order of 100 μm (*25*, *36*, *41*, *42*). However, the computational effort required to transform raw tactile images into three-dimensional (3D) contact maps inherently introduces latency, due to the multistage processing pipelines employed in vision-based tactile sensing. Such devices typically involve photometric calibration, illumination normalization, surface normal estimation (through photometric stereo techniques), normal integration for depth reconstruction, and subsequent spatial mapping, to obtain high-resolution 3D contact geometries and pressure distributions (*36*, *43*–*45*).

Here, we introduce a strategy to achieve an unprecedented combination of high resolution, real-time operation, and inherent simplicity in vision-based tactile sensing. It uses a stretchable mechanochromic elastomer that can convert mechanical strains into spatially resolved structural colors. So far, available mechanochromic materials (*46*, *47*) have been used in the form of dyes (e.g., spiropyrans and naphthopyrans) (*48*, *49*), liquid crystal elastomers (*50*), and photonic crystals (*51*) to investigate various applications, such as wearable motion/pressure sensors (*52*–*54*) and pressure sensors for minimally invasive surgery (*55*). However, their potential for enabling high-resolution and real-time tactile sensing in robotics remains largely unexplored.

The mechanochromic sensing concept proposed here is illustrated in Fig. 1C, in comparison with the two main vision-based tactile sensing strategies available to date (Fig. 1, A and B). The ability to encode mechanical stimuli into color patterns allows for direct transduction of information from the mechanical to the optical domain.

Figure 1D illustrates an example of an envisioned embodiment of this mechanochromic technology, where the sensor is embedded into the structure of an anthropomorphic robotic finger. A transparent elastomer is molded as a fingertip, integrating a camera and a light-emitting diode ring, and is coated with a bilayer structure consisting of a mechanochromic elastomer paired with a black elastomeric backing. A prototype implementation of this concept is shown in Fig. 1E, where a silicone fingertip was coated with a patch of the mechanochromic bilayer, which was fabricated as described in Materials and Methods. In the proof-of-concept real-time tactile sensing experiment shown in Fig. 1F, the robotic finger was pressed against the surface of a one-penny coin, allowing the soft tactile interface to conform to the coin’s topography and transduce local strains into color variations. The internal camera stream was used to directly read in real time from each pixel the hue value (as a quantification of the color), to visualize the contact spatial map as a 3D hue map (Fig. 1F). The following section provides details on the mechanochromic sensor structure, working principle, and tactile sensing performance.

## RESULTS

### Structure and working principle

**Fig. 2.**
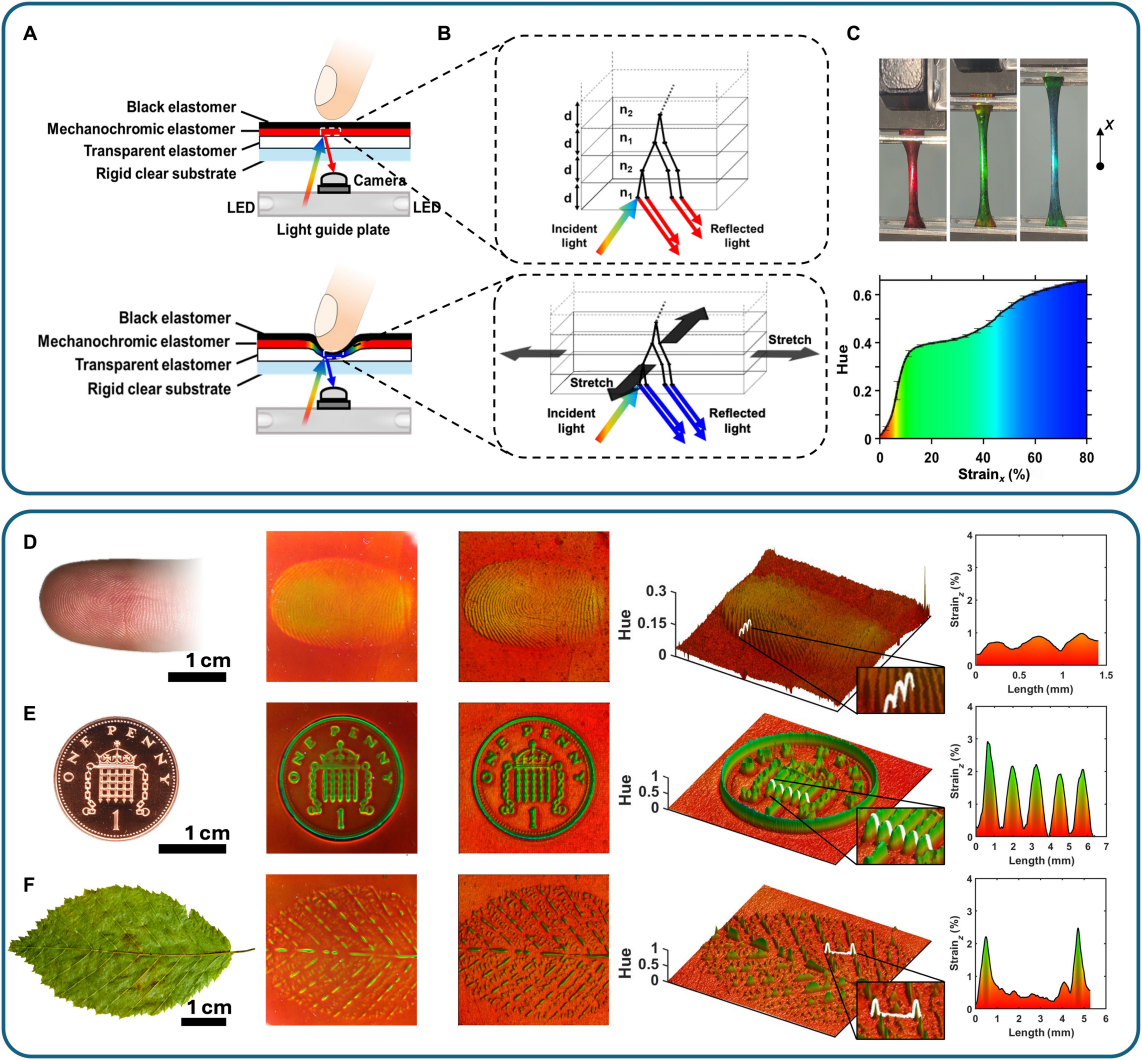
Mechanochromic tactile sensor: Structure, working principle, and examples of contact morphology maps. (**A**) Schematic representation of the structure of a possible implementation of the sensor, shown in its uncompressed (top) and compressed (bottom) states; in comparison to the general concept presented in Fig. 1C, here, the transparent elastomer was coupled to a rigid, clear substrate, for simplicity. (**B**) Illustration of the working principle of the mechanochromic material, as a stretchable Bragg reflector. (**C**) Uniaxial tensile testing of the mechanochromic elastomer, showing a strain-dependent shift in color, quantified as a variation in hue. (**D** to **F**) Tests of contact morphology mapping, where the sensor was pressed onto the fingertip, one-penny coin, and leaf that are shown on the left side. To enhance visualization of each object’s surface topography, the subsequent images visualize (from left to right): the mechanochromic raw picture captured by a digital single-lens reflex camera (EOS 1300D with a 18- to 55-mm lens, Canon, USA); the extracted 2D and 3D hue maps; a plot, along the indicated white line, of the perpendicular strain component in the mechanochromic layer (see the text for details).

The sensor comprises a mechanochromic elastomeric layer, sandwiched between two additional elastomeric layers: a black one, forming the sensor’s outer surface, and a transparent one, representing the inner part, facing the camera. To enhance the mechanochromic layer’s deformation (and, hence, change in color) when the sensor comes into contact with an external object, the three soft layers have to be coupled to a stiffer (preferably rigid), transparent substrate. This can be accomplished in various ways. For instance, in Fig. 1 (D and E), the substrate was the camera itself, embedded within the transparent elastomer. Alternatively, a separate rigid support, such as a glass plate, can be added, as shown in Fig. 2A. The latter is the configuration that was investigated in detail in this work.

In addition to the soft mechanochromic interface and its rigid support, the sensor includes a single camera, as well as a light guide plate generating diffused illumination. When an object is pressed against the sensor’s soft interface, any resulting strain of the mechanochromic layer is transduced into a spatially resolved structural color change, captured by the camera (Fig. 2A). The structural origin of the effect makes the response time limited only by the viscoelastic relaxation time constant of the constitutive elastomers, typically on the order of 1 ms or higher, making the transduction effectively quasi-instantaneous.

In this work, the mechanochromic elastomer was obtained from a 16-μm-thick photo-elastomeric film through a known photo-polymerization process (*56*), described in Materials and Methods. The resulting photonic material can respond to continuously varying mechanical stretches, by displaying a continuous color shift across the visible spectrum, from red to green and then to blue. The working principle is illustrated in Fig. 2B.

As a result of the photo-polymerization at a given exposure wavelength λ_exp_ (635 nm in this work), the material presents in its undeformed state a multilayered internal nanoscale structure, consisting of λ_exp_/4-thick parallel layers, which together form a periodic profile of refractive index (and density) *n* (Fig. 2B, top). This makes the mechanochromic elastomer behave as a stretchable Bragg reflector, such that incident light is partially transmitted and partially reflected at each internal nanoscale interface, where a change in density occurs (*46*, *47*, *57*). Owing to phase shifts (due to spatial propagation) of each reflected wave, constructive interference generates reflected light, having a peak wavelength λ_ref_ that obeys the general Bragg’s law

$$m\lambda_{\text{ref}}=2d\text{sinθ}$$ (1)

where d is the single nanolayer thickness, θ is the angle of incidence, equal to the angle of reflection, and m is the diffraction order (*m* = 1 in this case).

Any imposed deformation of the material that decreases its nanolayer thickness (Fig. 2B, bottom) causes a reduction in the reflected wavelength λ_ref_ (Eq. 1), leading to a change in the structural color observable with the camera. In particular, the variation of λ_ref_ can conveniently be related to the strain ε*_z_* resulting along the direction *z* perpendicular to the nanolayers, as follows

$$\lambda_{\text{ref}}=2d_{0}\left(1+\varepsilon_{z}\right)\text{sinθ}$$ (2)

The angular dependence of λ_ref_ implies that, for any given strain range, the broadest variation in color is observable from a perpendicular direction relative to the mechanochromic film plane. Accordingly, in this work, the camera was oriented perpendicularly to the sensor’s surface (Fig. 2A).

The distance between the camera and the sensor should be defined according to the characteristics of the adopted camera and lens, as well as the desired field of view (corresponding to the monitored sensing area). The black elastomer in Fig. 2A serves two key functions: It enhances color contrast and avoids optical interference from external light. The mechanochromic sensor can have complementary uses, to obtain accurate maps of contact morphology, strain, or pressure, as described below.

### Contact morphology and strain mapping

Detecting the morphology of the contact region between a tactile sensor’s soft interface and an object is crucial for a wide range of applications. For example, it can enable accurate mapping of the topography of textured surfaces, which is valuable for tactile inspections and object recognition, as illustrated in Fig. 1F. Another example is mapping the spatial distribution of contact points or areas, to be used as control feedback for robotic grasping and manipulation tasks. Depending on the intended function and the geometrical and mechanical properties of the contacted object, such applications may require contact morphology mapping at high spatial resolution.

The mechanochromic sensor’s achievable resolution is primarily determined by the following factors. The first one is the mechanochromic sensitivity of the system, which results from the combination of the intrinsic sensitivity of the mechanochromic elastomer and the spectral sensitivity of the camera. For instance, for the material used in this study (see Materials and Methods) in combination with a low-cost camera (HBVCAM-5M1966 V110, Bewinner, China), Fig. 2C shows the mechanochromic response, expressed as strain-induced color variations, quantified through changes in hue. The response covered the visible spectrum, from red to blue. Regions corresponding to the primary colors (red, green, and blue) generally exhibited plateaus of low sensitivity to strain (∼0.8 × 10^−3^%^−1^), calculated as the hue derivative relative to the strain component along the stretching direction (hereinafter referred to as Strain*_x_*). In contrast, transitions between red and green (yellow), and between green and blue (cyan), displayed steeper hue gradients, corresponding to a higher sensitivity (∼50.7 × 10^−3^%^−1^). Such a nonuniform sensitivity was substantially influenced by the camera’s spectral sensitivity, which, for low-end imaging devices, is typically higher at transitions between primary colors. A plot of the sensitivity to strain as a function of Strain*_x_* is presented in the fig. S1.

A second factor influencing the spatial resolution is the camera’s performance, not only in terms of spectral sensitivity (as highlighted above) but also in terms of optical resolution. These parameters offer design flexibility to optimize, for any given material, the mechanochromic response and thus the overall tactile-sensing accuracy. For instance, the compact, low-cost imaging system used in the robotic finger test (Fig. 1, E and F) was replaced by a larger and more sophisticated reflex camera in the experiments of Fig. 2 (D to F). While the mechanochromic sensor had the same structure (0.3-mm-thick black elastomer, 16-μm-thick mechanochromic elastomer, and 3-mm-thick transparent elastomer), the higher-performance camera allowed for capturing finer surface details, such as those of a human fingertip, a one-penny coin, and a leaf, with a spatial resolution of ∼100 μm, as evident from the topographic features in Fig. 2 (D to F).

A third factor determining the spatial resolution is the distance between the camera and the mechanochromic elastomeric layer. While increasing that distance expands the field of view, it also reduces the effective spatial resolution, as the same number of pixels in the photodetector should map a larger mechanochromic area.

A fourth factor that can notably affect the sensor’s spatial resolution is the geometry and mechanics of the contact with the object. For instance, in the experiments shown in Fig. 2 (D to F), the sensor was pressed against the objects manually, without any control over the contact force. The resulting resolution depended not only on the mechanochromic material’s sensitivity, and the camera’s spectral sensitivity, resolution, and distance but also on the complex interplay among the following mechanical and geometrical quantities: the applied contact pressure, the contact region size, the elastic properties of the sensor’s constitutive layers, and the thickness of each layer.

**Fig. 3.**
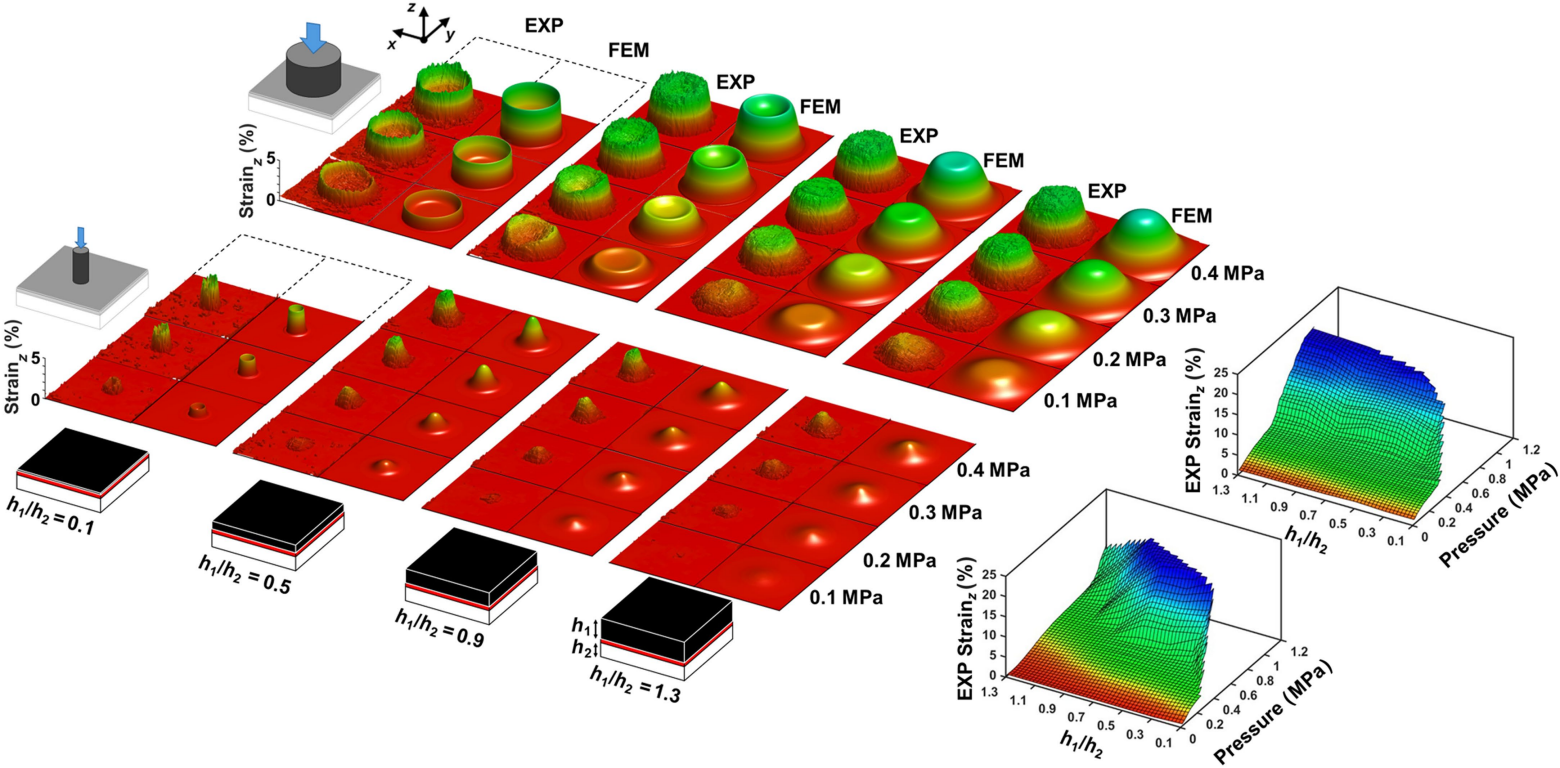
Contact morphology and strain mapping: Testing and FEM-based validation. Comparisons among the results of systematic indentation tests and corresponding finite element modeling (FEM)–based simulations. Strain*_z_* maps originated by indenting the sensor with 3-mm- and 10-mm-wide cylindrical indenters are shown for different contact pressures, applied to sensor configurations with different layer thickness ratios *h*_1_/*h*_2_. The left part of the figure shows a matrix visualization of representative experimental strain maps (EXPs) together with their corresponding FEM-based simulations. On the right side of the figure, two 3D interpolation plots, one for each indenter size, summarize the experimental results for all tested combinations of layer thickness ratio and applied pressure, illustrating their combined influence on the maximum Strain*_z_*. The tests were performed with a camera (HBVCAM-5M1966 V110, Bewinner, China) positioned 7 cm away from the mechanochromic interface. A video of the tests is available as movie S2.

To investigate these aspects, the sensor’s performance for strain and contact morphology mapping was systematically evaluated through comparative experiments. As detailed in Materials and Methods, the same constitutive elastomeric materials were used to manufacture the sensor in different configurations, which were then subjected to indentation tests, while varying the following quantities: the diameter of a flat cylindrical indenter (3 and 10 mm), the indentation pressure (up to ∼1 MPa), and the ratio between the black elastomer layer’s thickness *h*_1_ and the transparent elastomer layer’s thickness *h*_2_, hereinafter referred to as layer thickness ratio *h*_1_/*h*_2_. In particular, by varying *h*_1_ in the range 0.3 to 3.9 mm, while keeping *h*_2_ = 3 mm, the layer thickness ratio *h*_1_/*h*_2_ was varied in the range 0.1 to 1.3. The results are presented in Fig. 3.

To make the data visualization independent of the camera’s spectral sensitivity, the hue maps were converted into strain maps. These were obtained by combining the material’s hue-strain uniaxial characterization (Fig. 2C) with mechanical finite element modeling (FEM), as detailed in Materials and Methods. This provided an estimate of the through-thickness strain component in the sensor’s mechanochromic layer, hereinafter referred to as Strain*_z_*.

FEM was also used to simulate the sensor’s mechanical response to indentation, to validate its performance. To that end, Strain*_z_* maps resulting from mechanochromic sensing were compared to those obtained from FEM simulations (Fig. 3). The experimental strain map and FEM datasets had in each case a close correlation, as shown by the correlation plots presented in fig. S2, with a Pearson’s coefficient varying between 0.93 and 0.97; the root mean square error between the datasets, normalized by the strain range, varied between 8.2 and 10.9%.

Both the experimental results and the related simulations consistently showed the following evidence. In addition to the expected increase in strain with the applied pressure for each value of layer thickness ratio, a particularly noteworthy outcome was the effect of the mechanochromic elastomer layer’s depth relative to the contact surface on the overall spatial resolution and pressure sensitivity. Specifically, when the mechanochromic elastomer layer was positioned closer to the surface (i.e., at a low layer thickness ratio), the strain was primarily localized around the edges of the indentation area. In contrast, at greater depths (i.e., higher layer thickness ratios), the strain distribution became progressively more uniform, resulting in a gradual loss of edge definition and, consequently, a reduction in spatial resolution (Fig. 3).

Furthermore, the maps reveal that a higher layer thickness ratio led to a reduction in the sensor’s sensitivity to applied pressure. This effect was particularly evident in the experiments using the smaller indenter (Fig. 3), whose diameter was comparable to the total thickness of the sensor’s soft interface.

Both these effects directly resulted from depth-dependent strain fields produced by the indentation, which caused the mechanochromic elastomer to generate distinct chromatic patterns depending on its arrangement at different depths. These outcomes suggest that the sensor’s configuration can be tailored to match different functional requirements. In particular, low layer thickness ratios provide high spatial resolution, enabling detailed contact morphology mapping (Fig. 2), whereas higher ratios are better suited for assessing averaged pressures over confined regions (as is further investigated in the next section).

### Contact pressure mapping

Accurately mapping the spatial distribution of pressures applied to a tactile sensor can be valuable for various needs. For instance, it can improve control feedback for robotic grasping and manipulation, to reduce the risk of object damage or slippage, especially when interacting with delicate or irregularly shaped surfaces.

**Fig. 4.**
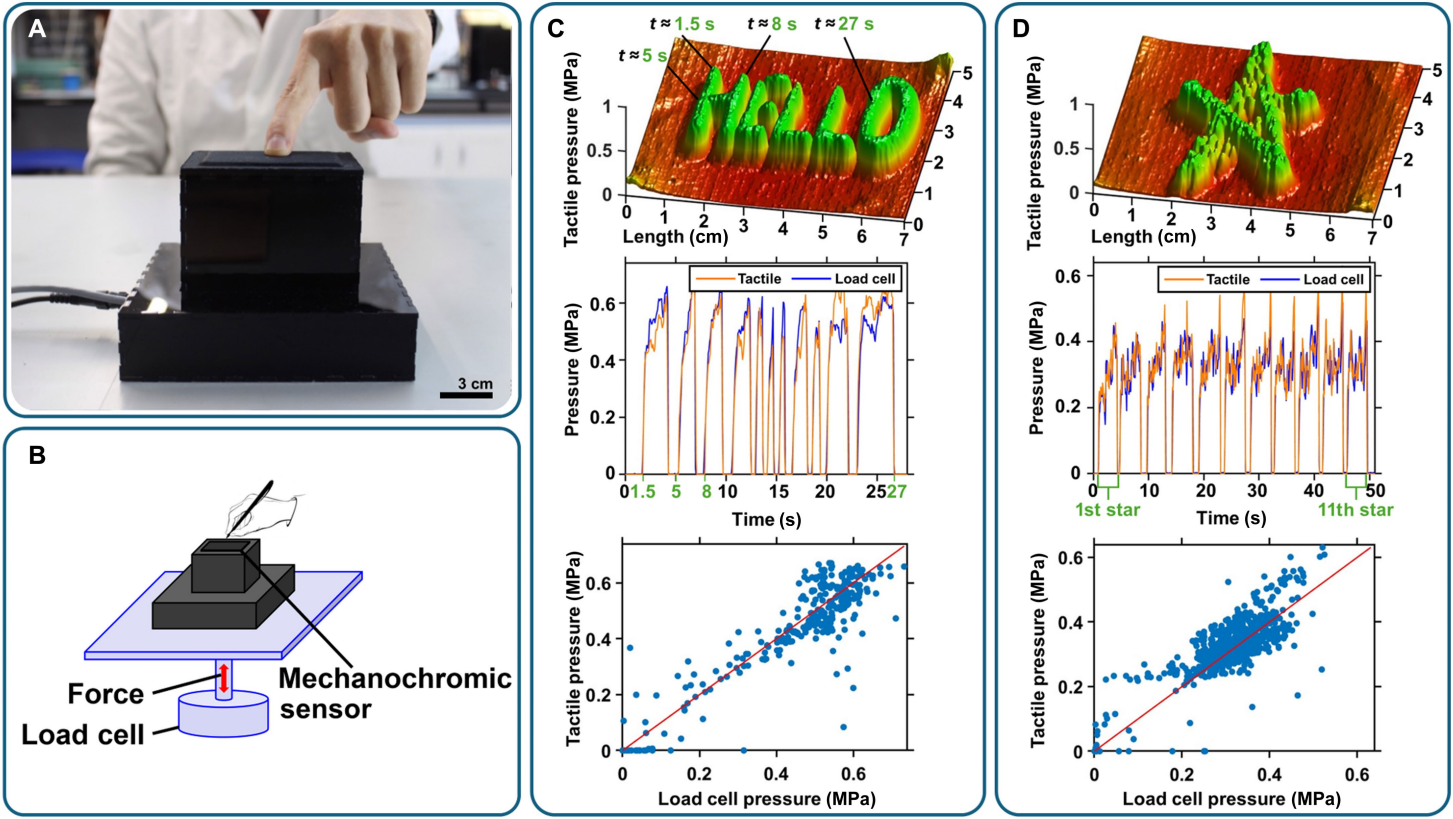
Contact pressure mapping: Testing and experimental validation. (**A**) Photograph of the mechanochromic sensor used as a writing and drawing surface, for pressure mapping tests. (**B**) Schematic of the experimental setup, where the sensor was mechanically coupled to a load cell. (**C**) Example of a writing test: a subject “wrote” on the sensor the word “HELLO.” The panels from top to bottom respectively show: the cumulative pressure map (obtained by combining sequential video frames to generate a composite image of the whole word); the related pressure signals captured by the mechanochromic sensor and the load cell (highlighted in green are the instants corresponding to sample marks on the pressure map); a correlation plot between the two pressure data sets. A video of the real-time pressure-sensing test is available as movie S3. (**D**) Example of a drawing test: The subject drew on the mechanochromic sensor the shape of a star, overlapping 11 consecutive repetitions. As for the writing test, the panels show the cumulative pressure map, the related pressure signals (highlighting in green the duration of the first and last drawing repetitions), and a correlation plot between the two pressure data sets. The tests were performed with a camera (HBVCAM-5M1966 V110, Bewinner, China) positioned 7 cm away from the sensor’s interface.

To investigate and demonstrate its pressure mapping performance, the mechanochromic tactile sensor was used as a writing and drawing surface, shown in Fig. 4A. The sensor was arranged on a plate, coupled to a load cell, according to the setup presented in Fig. 4B. The load cell allowed us to measure the force and calculate the pressure applied during writing and drawing, to validate pressure estimates inferred from the mechanochromic response.

A subject was asked to write and draw on the surface, using a cylindrical stylus having a 3-mm-wide flat circular tip. Its shape and size were selected to match those of the smaller indenter used for the indentation experiments.

The sensor was fabricated with a layer thickness ratio of 0.9, as this was the value that, when used with the smaller indenter, had produced a rather homogeneous pressure distribution (Fig. 3). This allowed us to use the indentation test data to calibrate the device as a pressure sensor, converting the captured hue maps into pressure maps. Pressure data derived from the calibrated mechanochromic response were validated by comparison with values obtained from the load cell, whose measured force was normalized by the stylus’ cross-sectional area.

The sensor exhibited a maximum sensitivity to pressure of ∼1.4 MPa^−1^. A plot of the sensitivity to pressure as a function of the pressure intensity is presented in the fig. S3.

Results of real-time pressure-sensing tests (writing and drawing) are shown in Fig. 4 (C and D). In particular, the top panels visualize cumulative pressure maps. They were obtained by overlaying sequential instantaneous maps and were used to ease the visualization of the writing/drawing process. The sensing dynamics can be appreciated from the pressure-time signals simultaneously captured by the tactile sensor and the load cell, reported in the middle panels. The two data sets had a close correlation, as shown by the correlation plots in the bottom panels, with a Pearson’s coefficient of ∼0.97 (writing test) and ∼0.93 (drawing test); the root mean square error between the datasets, normalized by the pressure range, was ∼9% (writing test) and ∼10% (drawing test).

An additional test was performed to assess the pressure-sensing accuracy at a single location, randomly chosen on the sensor’s surface. To that aim, the same stylus used for the tests described above was employed to press and release the sensor multiple times, with an arbitrarily increasing force. The results are presented in the fig. S4, which shows, as for the previous tests, a substantial correlation between the two sets of pressure detected by the sensor and the load cell. These findings indicate that the sensor’s response was accurately consistent with reference measurements from the load cell, validating the pressure-sensing capability.

## DISCUSSION

The mechanochromic tactile sensing technology presented herein exhibited outstanding performance in generating real-time spatial maps of contact morphology, strain, and pressure, achieving both high resolution and high accuracy. Unlike conventional tactile sensors that rely on discrete arrays of distinct mechanoelectrical taxels, this approach leverages a continuous sensing interface that inherently enables higher resolutions. Specifically, whereas state-of-the-art taxel-based sensors are unable to resolve features finer than the order of 1 mm, even when assisted by deep learning–based tactile super-resolution methods (*1*), the mechanochromic sensors readily enabled an order-of-magnitude improvement, showing a resolution of ∼100 μm. This performance is comparable to that of the most advanced vision-based tactile sensors that use directional light (*25*, *36*, *41*). However, that level of resolution was advantageously ensured by the mechanochromic sensor without the computational latency of key processing steps that are no longer required: surface normal estimation (photometric stereo), depth reconstruction, and spatial mapping. With the mechanochromic strategy, mechanical deformations are directly transduced into optical (colorimetric) maps, thereby eliminating the need for indirect 3D shape inference.

Notably, the high spatial resolution was also achieved without any data enhancement through deep learning–based image processing. In addition, no optimization was carried out at either the material level (e.g., elastic modulus, layer thickness, or mechanochromic sensitivity) or the camera level (e.g., photodetector resolution and spectral response). It is therefore reasonable to expect that targeted optimizations along these factors could further improve the achievable resolution. The material manufacturing does not rely on complex processing steps, potentially enabling straightforward industrial implementation with high scalability and throughput.

The sensor design can be tailored to address a variety of sensing requirements. Specifically, higher layer thickness ratios facilitate larger measurable ranges of contact pressure, whereas lower ratios improve spatial resolution and edge discrimination (Fig. 3). This design versatility makes mechanochromic tactile sensing potentially valuable across a wide range of applications. For example, precise monitoring of the spatial distribution of contact forces can benefit robotic grasping and manipulation, as well as enhance human-robot interactions. Another possible application lies in high-resolution tactile mapping for automated product inspection, allowing the identification of surface defects or damage in contexts where visual inspection is challenging or infeasible, such as with transparent or highly reflective materials.

Future developments of this technology may address the following aspects. The structural colors generated by the mechanochromic elastomer’s deformations are angle dependent (iridescence), as quantified by Eq. 2. This implies that calibrations performed along any specific direction (such as orthogonal to the sensor surface, as done in this work) would tend to lose accuracy as the optical information reaches the camera at increasing angles. As a consequence, accuracy would tend to progressively decrease with increasing sensing area and/or curvature. However, if the distance between the mechanochromic elastomer layer and the camera is progressively increased, effectively reducing the viewing angle at which the camera observes the sensing area, the loss of accuracy due to iridescence tends to become negligible (as was the case in the tests conducted in this work). On the other hand, reducing the distance between the camera and the sensor’s outer surface is desirable, to ensure compact form factors. Therefore, as the camera is positioned closer to the surface, compensation strategies to mitigate iridescence become increasingly important. While efforts to develop noniridescent mechanochromic elastomers capable of generating structural colors are ongoing (*58*–*60*), a straightforward approach would be to perform angle-dependent calibrations.

Compact form factors are of particular interest to facilitate integration into products. We anticipate that an optimized optical design (camera and lens) could substantially reduce the overall vertical size. In particular, the use of wide-angle lenses would allow the camera to be positioned closer to the surface. However, such lenses could also introduce drawbacks, such as angle-dependent color shifts, chromatic aberration, and reduced image contrast, which might lower the effective spatial resolution, especially near the edges of the field of view. This would imply the need for a trade-off between vertical size and peripheral tactile sensing accuracy. Nevertheless, such a limitation might be mitigated with ad hoc compensation strategies, such as angle-dependent corrections to the calibration.

Future investigations should also include cyclic fatigue testing, to assess the durability of the mechanochromic bilayer under repeated loading (according to application-driven requirements), as well as possible hysteretic effect in the hue-strain relationship. Nevertheless, as the color-changing effect has a structural origin, the durability is expected to be consistent with the elastomeric nature of the constitutive materials.

Overall, we anticipate that the seamless applicability of this mechanochromic strategy to enhance vision-based tactile sensing in robotics will open a transformative path, enabling a unique combination of real-time responsiveness, high spatial resolution, and inherent simplicity.

## MATERIALS AND METHODS

### Mechanochromic sensor manufacturing

The main component of the tactile sensor was the mechanochromic elastomeric layer, which was obtained as follows. A 16-μm-thick photo-elastomeric film (C-RT20, Litiholo, USA) was processed according to the Lippmann photographic technique. In a darkroom environment, the film was layered on a stainless-steel shim stock, serving as a reflecting substrate with a smooth surface, and was exposed to a 5-mW red (635 nm wavelength) LASER light (Litiholo, USA). The light was applied to the sample for 7 min, from a distance of 30 cm, according to the manufacturer’s instructions. The LASER beam reflection from the backing layer created an interference pattern in the form of a standing wave across the photo-elastomeric film thickness. The standing wave was able to generate within the photo-elastomeric film a spatially differentiated photo-polymerization, leading to a modulation of the material’s density that mirrored the periodicity of the interference pattern. A schematic drawing is presented in the fig. S5. As a result, the photo-elastomeric film developed an internal microscale layering, which imparted mechanochromic properties, arising from a deformation-dependent structural coloration.

To ensure bonding with the black and transparent elastomeric layers, the photo-elastomeric film was plasma-treated in a plasma oven (Zepto, Diener Electronic, Germany) for 90 s at full power. The black elastomeric layer was obtained as follows. A polydimethylsiloxane (PDMS) silicone elastomer (Sylgard 184, Dow Inc., USA) was processed with its cross-linker at a 1:10 ratio and then mixed with 30 wt % of a black dye (SilcPig, Smooth-On Inc., USA). The resulting mixture was layered onto the plasma-treated mechanochromic film, using the doctor blade technique, and then cured in an oven at 80°C for 24 hours. Its thickness was varied according to the range of test geometries investigated, as specified in the main text.

The sensor’s structure was then completed with a 3-mm-thick layer of the Sylgard 184 PDMS transparent elastomer, which was processed with a 1:10 cross-linker ratio and cured at 80°C for 48 hours. The obtained three-layer soft structure was arranged onto a 3-mm-thick transparent glass plate.

### Elastomers uniaxial tensile testing

The mechanical stress-strain properties of the mechanochromic elastomer, the black elastomer and the transparent elastomer were separately characterized with uniaxial tensile tests, using a mechanical testing machine (68TM-10, Instron, USA) equipped with a video extensometer (AVE3, Instron, USA). Each sample was dog-bone shaped, marked with two visible tracking dots for automated strain measurement, and tested at a strain rate of 50 mm/min. The sample dimensions were as follows: The black elastomer and the transparent elastomer had a length (distance between the two tracking dots) of 9.5 mm, a width of 3 mm, and a thickness of 0.3 mm; the mechanochromic elastomer had a length (distance between the two tracking dots) of 10 mm, a width of 4 mm, and a thickness of 0.016 mm.

### Mechanochromic bilayer characterization

The chromatic response to strain of the mechanochromic bilayer (i.e., the mechanochromic elastomer layer coupled to the black elastomer layer) was investigated by measuring variations in hue during uniaxial tensile testing. Six dog-bone-shaped specimens were prepared by laminating a 300-μm-thick layer of the black elastomer onto the 16-μm-thick photonic elastomer film, previously processed as described above. The specimens were stretched using a mechanical testing machine (5900R-84, Instron, UK) at a constant speed of 20 mm/min, until material failure.

The mechanochromic response was characterized using a USB camera (HBVCAM-5M1966 V110, Bewinner, China) mounted perpendicularly to the specimen, under constant illumination. The video recording was synchronized with the mechanical recording through a MATLAB script, which used a trigger signal received from the mechanical testing machine. The video frame sequence was processed by extracting, for any applied strain, the corresponding hue as the average value from the pixels of a central rectangular region. The measured hue was then further averaged among the six specimens. As a result, the process returned the overall hue-strain relationship, shown in Fig. 2C, where the error bars correspond to the standard deviation among all the samples.

### Mechanochromic sensor indentation testing

Indentation experiments were performed using a mechanical testing machine (5900R-84, Instron, UK) at a constant speed of 1.5 mm/min. Two flat cylindrical indenters with diameters of 3 and 10 mm were comparatively used to apply indentation pressures up to ∼1 MPa, perpendicularly to the sensor’s surface. Comparative tests were performed on different samples of the sensor, which were manufactured with different values of the layer thickness ratio *h*_1_/*h*_2_. While the transparent elastomer layer’s thickness *h*_2_ was held constant (3 mm) across all samples, the black elastomer layer’s thickness *h*_1_ was varied among seven values (0.3, 0.9, 1.5, 2.1, 2.7, 3.3, and 3.9 mm), respectively corresponding to the following values of layer thickness ratio *h*_1_/*h*_2_: 0.1, 0.3, 0.5, 0.7, 0.9, 1.1, and 1.3. To minimize friction during testing, talc powder was applied to the sensor’s surface in contact with the indenter.

Color variations induced by indentation were detected using a USB camera (HBVCAM-5M1966 V110, Bewinner, China) mounted perpendicularly to the sample, under constant illumination. The recorded hue values were converted into Strain*_z_* values (as described in the main text).

### FEM

FEM was performed using a commercial software (ABAQUS CAE 2023, Dassault Systèmes SIMULIA, France) to obtain simulated Strain*_z_* maps in the mechanochromic elastomeric layer during indentation. A block diagram of the FEM workflow is shown in the fig. S6. All constitutive materials were modelled as hyperelastic, using the built-in Yeoh material model provided by ABAQUS. The model parameters obtained by fitting the experimental stress-strain curves are reported in the table S1. FEM settings are specified in the table S2.

FEM was also performed for the mechanochromic bilayer to establish a numerical relationship between Strain_*x*_ and Strain_*z*_. This relationship, together with the experimentally determined dependence of hue on Strain_*x*_, enabled us to convert the hue maps obtained during indentations into Strain_*z*_ maps. The model developed for this purpose was meshed with 3D stress elements (C3D8RH). The best-fit relationship between Strain*_x_* and Strain*_z_* was expressed as a third-order polynomial

$${\text{Strain}}_{z}=-0.0945\;{{\text{Strain}}_{x}}^{3}+0.2639\;{{\text{Strain}}_{x}}^{2}-0.3848\;{\text{Strain}}_{x}$$ (3)

## References

[1] D. Kong, Y. Lu, S. Zhou, M. Wang, G. Pang, B. Wang, L. Chen, X. Huang, H. Lyu, K. Xu, G. Yang, Super-resolution tactile sensor arrays with sparse units enabled by deep learning. Sci. Adv. 11, eadv2124 (2025). 40601743 10.1126/sciadv.adv2124 PMC12219507

[2] A. Chortos, J. Liu, Z. Bao, Pursuing prosthetic electronic skin. Nat. Mater. 15, 937–950 (2016). 27376685 10.1038/nmat4671

[3] Z. Ye, G. Pang, K. Xu, Z. Hou, H. Lyu, Y. Shen, G. Yang, Soft robot skin with conformal adaptability for on-body tactile perception of collaborative robots. IEEE Robot. Autom. Lett. 7, 5127–5134 (2022).

[4] R. De Fazio, V. M. Mastronardi, M. Petruzzi, M. De Vittorio, P. Visconti, Human–machine interaction through advanced haptic sensors: A piezoelectric sensory glove with edge machine learning for gesture and object recognition. Future Internet 15, 14 (2023).

[5] J. Xu, J. Pan, T. Cui, S. Zhang, Y. Yang, T.-L. Ren, Recent progress of tactile and force sensors for human–machine interaction. Sensors 23, 1868 (2023). 36850470 10.3390/s23041868 PMC9961639

[6] J. O’Neill, J. Lu, R. Dockter, T. Kowalewski, Stretchable, flexible, scalable smart skin sensors for robotic position and force estimation. Sensors 18, 953 (2018). 29570643 10.3390/s18040953 PMC5948948

[7] S. Luo, J. Bimbo, R. Dahiya, H. Liu, Robotic tactile perception of object properties: A review. Mechatronics 48, 54–67 (2017).

[8] J. Yang, S. Luo, X. Zhou, J. Li, J. Fu, W. Yang, D. Wei, Flexible, tunable, and ultrasensitive capacitive pressure sensor with microconformal graphene electrodes. ACS Appl. Mater. Interfaces 11, 14997–15006 (2019). 30869860 10.1021/acsami.9b02049

[9] Q. Su, Q. Zou, Y. Li, Y. Chen, S.-Y. Teng, J. T. Kelleher, R. Nith, P. Cheng, N. Li, W. Liu, S. Dai, Y. Liu, A. Mazursky, J. Xu, L. Jin, P. Lopes, S. Wang, A stretchable and strain-unperturbed pressure sensor for motion interference-free tactile monitoring on skins. Sci. Adv. 7, eabi4563 (2021). 34818045 10.1126/sciadv.abi4563 PMC8612682

[10] A. Rana, J.-P. Roberge, V. Duchaine, An improved soft dielectric for a highly sensitive capacitive tactile sensor. IEEE Sens. J. 16, 7853–7863 (2016).

[11] J. Zhang, S. Wei, C. Liu, C. Shang, Z. He, Y. Duan, Z. Peng, Porous nanocomposites with enhanced intrinsic piezoresistive sensitivity for bioinspired multimodal tactile sensors. Microsyst. Nanoeng. 10, 19 (2024). 38283382 10.1038/s41378-023-00630-z PMC10811241

[12] M. Jing, J. Zhou, P. Zhang, D. Hou, J. Shen, J. Tian, W. Chen, Porous AgNWs/poly(vinylidene fluoride) composite-based flexible piezoresistive sensor with high sensitivity and wide pressure ranges. ACS Appl. Mater. Interfaces 14, 55119–55129 (2022). 36451588 10.1021/acsami.2c17879

[13] S. B. Choi, T. Noh, S. Jung, J. Kim, Stretchable piezoresistive pressure sensor array with sophisticated sensitivity, strain-insensitivity, and reproducibility. Adv. Sci. 11, 2405374 (2024). 10.1002/advs.202405374 PMC11425275 39013112

[14] W. Lin, B. Wang, G. Peng, Y. Shan, H. Hu, Z. Yang, Skin-inspired piezoelectric tactile sensor array with crosstalk-free row+column electrodes for spatiotemporally distinguishing diverse stimuli. Adv. Sci. 8, 2002817 (2021). 10.1002/advs.202002817 PMC7856889 33552864

[15] J. Zhang, H. Yao, J. Mo, S. Chen, Y. Xie, S. Ma, R. Chen, T. Luo, W. Ling, L. Qin, Z. Wang, W. Zhou, Finger-inspired rigid-soft hybrid tactile sensor with superior sensitivity at high frequency. Nat. Commun. 13, 5076 (2022). 36038557 10.1038/s41467-022-32827-7 PMC9422944

[16] J. Yu, G. Gao, J. Huang, X. Yang, J. Han, H. Zhang, Y. Chen, C. Zhao, Q. Sun, Z. L. Wang, Contact-electrification-activated artificial afferents at femtojoule energy. Nat. Commun. 12, 1581 (2021). 33707420 10.1038/s41467-021-21890-1 PMC7952391

[17] B. Shao, M.-H. Lu, T.-C. Wu, W.-C. Peng, T.-Y. Ko, Y.-C. Hsiao, J.-Y. Chen, B. Sun, R. Liu, Y.-C. Lai, Large-area, untethered, metamorphic, and omnidirectionally stretchable multiplexing self-powered triboelectric skins. Nat. Commun. 15, 1238 (2024). 38336848 10.1038/s41467-024-45611-6 PMC10858173

[18] Y. Yan, Z. Hu, Z. Yang, W. Yuan, C. Song, J. Pan, Y. Shen, Soft magnetic skin for super-resolution tactile sensing with force self-decoupling. Sci. Robot. 6, eabc8801 (2021). 34043530 10.1126/scirobotics.abc8801

[19] Y. Wu, Y. Liu, Y. Zhou, Q. Man, C. Hu, W. Asghar, F. Li, Z. Yu, J. Shang, G. Liu, M. Liao, R.-W. Li, A skin-inspired tactile sensor for smart prosthetics. Sci. Robot. 3, eaat0429 (2018). 33141753 10.1126/scirobotics.aat0429

[20] C. Wang, L. Dong, D. Peng, C. Pan, Tactile sensors for advanced intelligent systems. Adv. Intell. Syst. 1, 1900090 (2019).

[21] D. Xu, A. Tairych, I. A. Anderson, Stretch not flex: Programmable rubber keyboard. Smart Mater. Struct. 25, 015012 (2016).

[22] E. Judd, K. M. Digumarti, J. Rossiter, H. Hauser, NeatSkin: A discrete impedance tomography skin sensor, in *2020 3rd IEEE International Conference on Soft Robotics (RoboSoft),* (IEEE, New Haven, CT, USA, 2020; https://ieeexplore.ieee.org/document/9115979/), pp. 33–38.

[23] D. Hardman, T. G. Thuruthel, F. Iida, Multimodal information structuring with single-layer soft skins and high-density electrical impedance tomography. Sci. Robot. 10, eadq2303 (2025). 40498812 10.1126/scirobotics.adq2303

[24] K. Park, H. Yuk, M. Yang, J. Cho, H. Lee, J. Kim, A biomimetic elastomeric robot skin using electrical impedance and acoustic tomography for tactile sensing. Sci. Robot. 7, eabm7187 (2022). 35675452 10.1126/scirobotics.abm7187

[25] H. Li, Y. Lin, C. Lu, M. Yang, E. Psomopoulou, N. F. Lepora, Classification of vision-based tactile sensors: A review. IEEE Sens. J. 25, 35672–35686 (2025).

[26] K. Shimonomura, Tactile image sensors employing camera: A review. Sensors 19, 3933 (2019). 31547285 10.3390/s19183933 PMC6767299

[27] C. Sferrazza, R. D’Andrea, Design, motivation and evaluation of a full-resolution optical tactile sensor. Sensors 19, 928 (2019). 30813292 10.3390/s19040928 PMC6412824

[28] N. F. Lepora, Y. Lin, B. Money-Coomes, J. Lloyd, DigiTac: A DIGIT-TacTip hybrid tactile sensor for comparing low-cost high-resolution robot touch. IEEE Robot. Autom. Lett. 7, 9382–9388 (2022).

[29] T. Assaf, C. Roke, J. Rossiter, T. Pipe, C. Melhuish, Seeing by touch: Evaluation of a soft biologically-inspired artificial fingertip in real-time active touch. Sensors 14, 2561–2577 (2014). 24514881 10.3390/s140202561 PMC3958268

[30] M. Li, T. Li, Y. Jiang, Marker displacement method used in vision-based tactile sensors—From 2-D to 3-D: A review. IEEE Sens. J. 23, 8042–8059 (2023).

[31] B. Ward-Cherrier, N. Pestell, L. Cramphorn, B. Winstone, M. E. Giannaccini, J. Rossiter, N. F. Lepora, The TacTip family: Soft optical tactile sensors with 3D-printed biomimetic morphologies. Soft Robot. 5, 216–227 (2018). 29297773 10.1089/soro.2017.0052 PMC5905869

[32] M. Lambeta, T. Wu, A. Sengul, V. R. Most, N. Black, K. Sawyer, R. Mercado, H. Qi, A. Sohn, B. Taylor, N. Tydingco, G. Kammerer, D. Stroud, J. Khatha, K. Jenkins, K. Most, N. Stein, R. Chavira, T. Craven-Bartle, E. Sanchez, Y. Ding, J. Malik, R. Calandra, Digitizing touch with an artificial multimodal fingertip. arXiv:2411.02479 [cs.RO] (2024).

[33] M. Lambeta, P.-W. Chou, S. Tian, B. Yang, B. Maloon, V. R. Most, D. Stroud, R. Santos, A. Byagowi, G. Kammerer, D. Jayaraman, R. Calandra, DIGIT: A novel design for a low-cost compact high-resolution tactile sensor with application to in-hand manipulation. IEEE Robot. Autom. Lett. 5, 3838–3845 (2020).

[34] S. Suresh, H. Qi, T. Wu, T. Fan, L. Pineda, M. Lambeta, J. Malik, M. Kalakrishnan, R. Calandra, M. Kaess, J. Ortiz, M. Mukadam, NeuralFeels with neural fields: Visuotactile perception for in-hand manipulation. Sci. Robot. 9, eadl0628 (2024). 39536124 10.1126/scirobotics.adl0628

[35] C. Lin, H. Zhang, J. Xu, L. Wu, H. Xu, 9DTact: A compact vision-based tactile sensor for accurate 3D shape reconstruction and generalizable 6D force estimation. IEEE Robot. Autom. Lett. 9, 923–930 (2024).

[36] W. Yuan, S. Dong, E. H. Adelson, GelSight: High-resolution robot tactile sensors for estimating geometry and force. Sensors 17, 2762 (2017). 29186053 10.3390/s17122762 PMC5751610

[37] S. Q. Liu, L. Z. Yañez, E. H. Adelson, GelSight endoflex: A soft endoskeleton hand with continuous high-resolution tactile sensing, in *2023 IEEE International Conference on Soft Robotics (RoboSoft),* (IEEE, Singapore, Singapore, 2023; https://ieeexplore.ieee.org/document/10122053/), pp. 1–6.

[38] W. Fan, H. Li, W. Si, S. Luo, N. Lepora, D. Zhang, ViTacTip: Design and verification of a novel biomimetic physical vision-tactile fusion sensor, in *2024 IEEE International Conference on Robotics and Automation (ICRA)*, (IEEE, Yokohama, Japan, 2024; https://ieeexplore.ieee.org/document/10611186/), pp. 1056–1062.

[39] I. H. Taylor, S. Dong, A. Rodriguez, GelSlim 3.0: High-resolution measurement of shape, force and slip in a compact tactile-sensing finger, in *2022 International Conference on Robotics and Automation (ICRA)*, (IEEE, Philadelphia, PA, USA, 2022; https://ieeexplore.ieee.org/document/9811832/), pp. 10781–10787.

[40] Z. Zhao, Y. Li, W. Li, Z. Qi, L. Ruan, Y. Zhu, K. Althoefer, Tac-Man: Tactile-informed prior-free manipulation of articulated objects. IEEE Trans. Robot. 41, 538–557 (2025).

[41] H. Sun, K. J. Kuchenbecker, G. Martius, A soft thumb-sized vision-based sensor with accurate all-round force perception. Nat. Mach. Intell. 4, 135–145 (2022).

[42] S. Li, Z. Wang, C. Wu, X. Li, S. Luo, B. Fang, F. Sun, X.-P. Zhang, W. Ding, When Vision meets touch: A contemporary review for visuotactile sensors from the signal processing perspective. IEEE J. Sel. Top. Signal Process. 18, 267–287 (2024).

[43] M. K. Johnson, E. H. Adelson, Retrographic sensing for the measurement of surface texture and shape, in *2009 IEEE Conference on Computer Vision and Pattern Recognition*, (IEEE, Miami, FL, 2009; https://ieeexplore.ieee.org/document/5206534/), pp. 1070–1077.

[44] J. Li, S. Dong, E. H. Adelson, End-to-end pixelwise surface normal estimation with convolutional neural networks and shape reconstruction using GelSight sensor, in *2018 IEEE International Conference on Robotics and Biomimetics (ROBIO)* (IEEE, Kuala Lumpur, Malaysia, 2018; https://ieeexplore.ieee.org/document/8665351/), pp. 1292–1297.

[45] U. H. Shah, R. Muthusamy, D. Gan, Y. Zweiri, L. Seneviratne, On the design and development of vision-based tactile sensors. J. Intell. Robot. Syst. 102, 82 (2021).

[46] C. Tang, Fundamental aspects of stretchable mechanochromic materials: Fabrication and characterization. Materials 17, 3980 (2024). 39203158 10.3390/ma17163980 PMC11355797

[47] G. Chen, W. Hong, Mechanochromism of structural-colored materials. Adv. Opt. Mater. 8, 2000984 (2020).

[48] F. Ciardelli, G. Ruggeri, A. Pucci, Dye-containing polymers: Methods for preparation of mechanochromic materials. Chem. Soc. Rev. 42, 857–870 (2013). 23188066 10.1039/c2cs35414d

[49] M. J. Robb, T. A. Kim, A. J. Halmes, S. R. White, N. R. Sottos, J. S. Moore, Regioisomer-specific mechanochromism of naphthopyran in polymeric materials. J. Am. Chem. Soc. 138, 12328–12331 (2016). 27616572 10.1021/jacs.6b07610

[50] P. Zhang, L. T. De Haan, M. G. Debije, A. P. H. J. Schenning, Liquid crystal-based structural color actuators. Light Sci. Appl. 11, 248 (2022). 35931672 10.1038/s41377-022-00937-y PMC9356073

[51] P. Zhao, B. Li, Z. Tang, Y. Gao, H. Tian, H. Chen, Stretchable photonic crystals with periodic cylinder shaped air holes for improving mechanochromic performance. Smart Mater. Struct. 28, 075037 (2019).

[52] T. Wang, L. Chen, H. Liu, H. Zhu, Z. Zeng, Y. Lu, P. Zhang, Y. Chen, Y. Huang, G.-S. Liu, Y. Luo, Z. Chen, Ultrasensitive bionic photonic-electronic skin with wide red-shift mechanochromic response. Light Adv. Manuf. 6, 20 (2025).

[53] X. Li, Y. Yang, C. Valenzuela, X. Zhang, P. Xue, Y. Liu, C. Liu, L. Wang, Mechanochromic and conductive chiral nematic nanostructured film for bioinspired ionic skins. ACS Nano 17, 12829–12841 (2023). 37338401 10.1021/acsnano.3c04199

[54] H. Zhang, H. Chen, J.-H. Lee, E. Kim, K.-Y. Chan, H. Venkatesan, X. Shen, J. Yang, J.-K. Kim, Mechanochromic optical/electrical skin for ultrasensitive dual-signal sensing. ACS Nano 17, 5921–5934 (2023). 36920071 10.1021/acsnano.3c00015

[55] G. Giordano, M. Gagliardi, Y. Huan, M. Carlotti, A. Mariani, A. Menciassi, E. Sinibaldi, B. Mazzolai, Toward mechanochromic soft material-based visual feedback for electronics-free surgical effectors. Adv. Sci. 8, e2100418 (2021). 10.1002/advs.202100418 PMC8336492 34075732

[56] B. H. Miller, H. Liu, M. Kolle, Scalable optical manufacture of dynamic structural colour in stretchable materials. Nat. Mater. 21, 1014–1018 (2022). 35915162 10.1038/s41563-022-01318-x

[57] J. M. Clough, C. Weder, S. Schrettl, Mechanochromism in structurally colored polymeric materials. Macromol. Rapid Commun. 42, e2000528 (2021). 33210385 10.1002/marc.202000528

[58] H. Tan, Q. Lyu, Z. Xie, M. Li, K. Wang, K. Wang, B. Xiong, L. Zhang, J. Zhu, Metallosupramolecular photonic elastomers with self-healing capability and angle-independent color. Adv. Mat. 31, e1805496 (2019). 10.1002/adma.201805496 30548887

[59] Y. Qi, C. Zhou, S. Zhang, Z. Zhang, W. Niu, S. Wu, W. Ma, B. Tang, Bar-coating programmable mechanochromic bilayer PDMS film with angle-dependent and angle-independent structural colors. Dyes Pigm. 189, 109264 (2021).

[60] Z. Wang, J. Zhang, Z. Lan, Q. Meng, R. Tang, X. Shen, Q. Sun, Stretchable photonic semicrystal interface by pressure-assistant self-assembly. Adv. Mater. Inter. 9, 2102047 (2022).

