# Supplementary material for: High-resolution real-time mechanochromic tactile sensors
Source: Sci Adv. 2026 Jul 3;12(27):eaee5236. doi: 10.1126/sciadv.aee5236 (PMC13330820; doi:10.1126/sciadv.aee5236)
Supplement: Supplementary file 1 — Figs. S1 to S6 Tables S1 and S2 Legends for movies S1 to S3 [file sciadv.aee5236_sm.pdf]

Science Advances
AAAS

Supplementary Materials for

High-resolution real-time mechanochromic tactile sensors

Giacomo Sasso *et al.*

Corresponding author: Giacomo Sasso, g.sasso@qmul.ac.uk; James J. C. Busfield, j.busfield@qmul.ac.uk; Federico Carpi, federico.carpi@unifi.it

Sci. Adv. **12**, eaee5236 (2026)
DOI: 10.1126/sciadv.aee5236

The PDF file includes:

Figs. S1 to S6
Tables S1 and S2
Legends for movies S1 to S3

Other Supplementary Material for this manuscript includes the following:

Movies S1 to S3

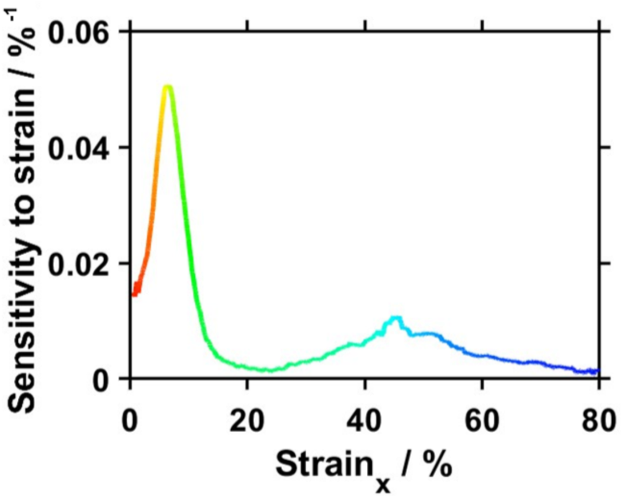

Fig. S1. Mechanochromic bilayer sensitivity to strain.

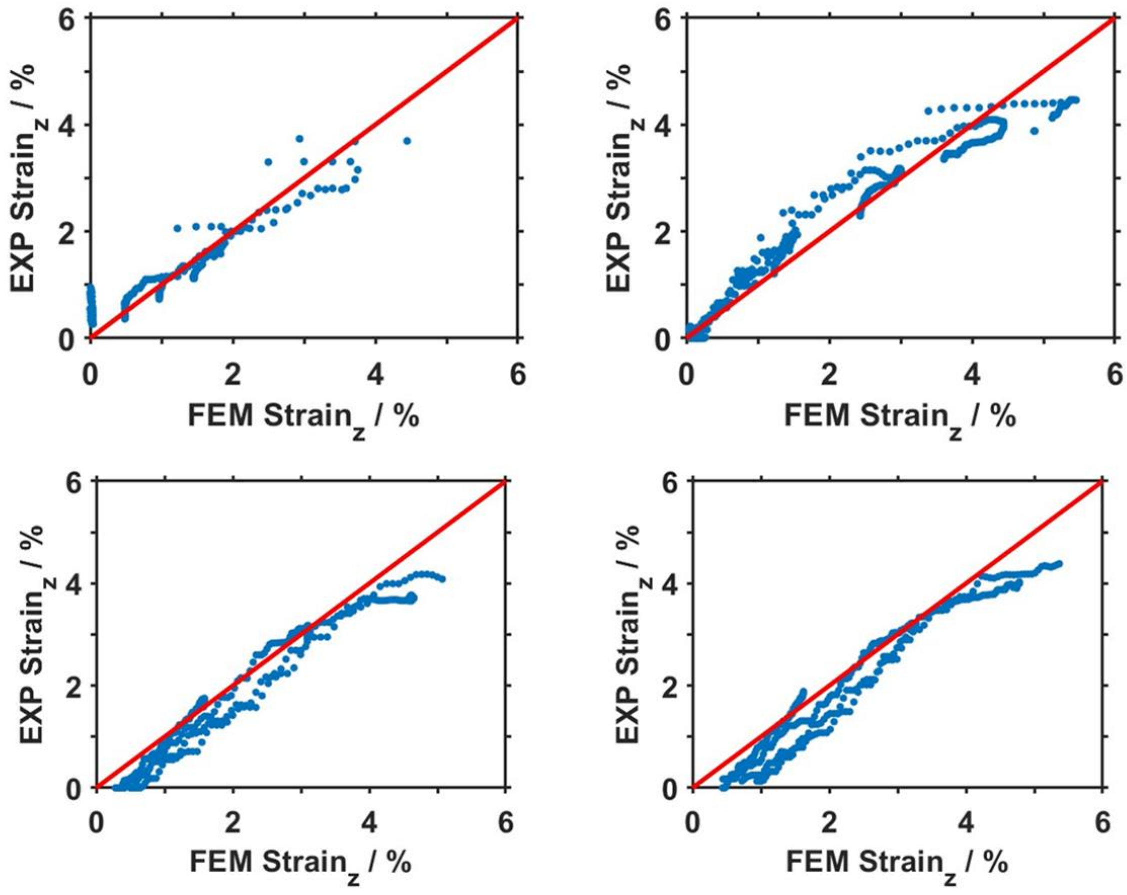

Fig. S2. Correlation plots between experimental strain and finite element model-based strain. The strains refer to the tests presented in Fig. 3, where different sensor configurations with a layer thickness ratio of 0.1 (**A**), 0.5 (**B**), 0.9 (**C**) and 1.3 (**D**) were indented with a 10mm-wide indenter, both experimentally (EXP) and with finite element model-based simulations (FEM). Each correlation plot is a collection of data extracted from the strain profiles obtained by sectioning all the 3D maps shown in Fig. 3 with an *xz* plane passing through the centre of the indentation area.

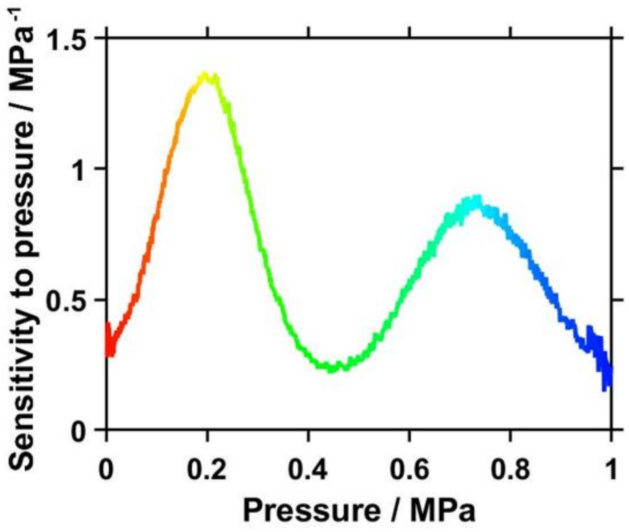

Fig. S3. Mechanochromic sensor sensitivity to pressure.

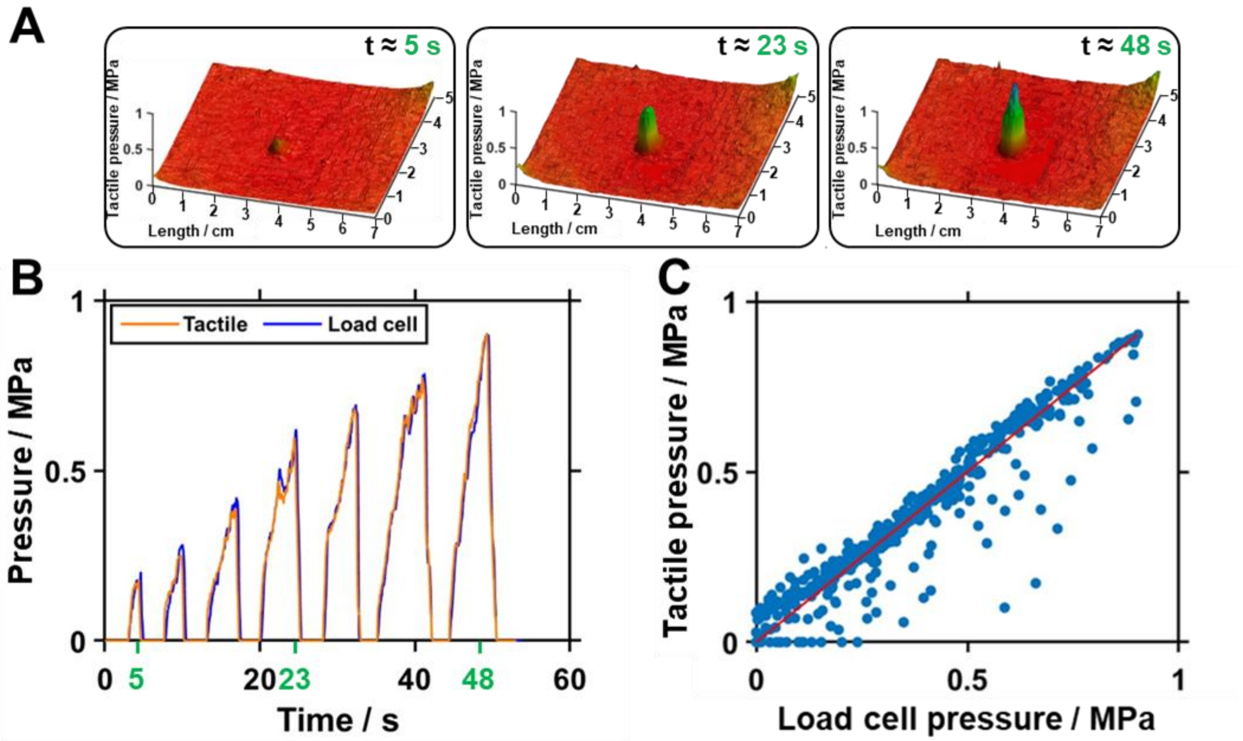

Fig. S4. Contact pressure sensing and validation. The same setup shown in the main text was used to assess the device's pressure-sensing accuracy at a single location. (**A**) Examples of real-time pressure maps detected by the sensor at different instants when a subject applied increasing loads (~0.2 MPa, ~0.55 MPa and ~0.9 MPa) at a randomly chosen location of the mechanochromic tactile sensor's surface. (**B**) Pressure-time signals captured by the tactile sensor and load cell, when the subject was asked to apply a sequence of seven increasing loads; highlighted in green are the instants corresponding to the sample images presented above. (**C**) Correlation plot between the two pressure sets.

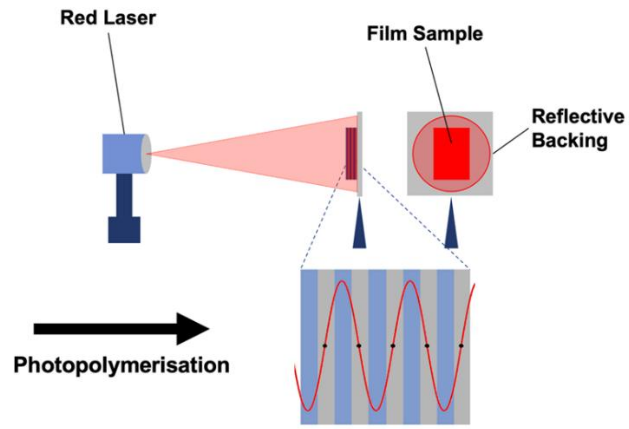

Fig. S5. Optical processing of the photo-elastomeric film to make it mechanochromic. The photo-elastomeric film was coupled to a reflective backing and was illuminated with a LASER light. A standing wave resulting in the film was able to induce a spatially alternating photo-polymerisation, producing alternating layers of high density (blue) and low density (grey).

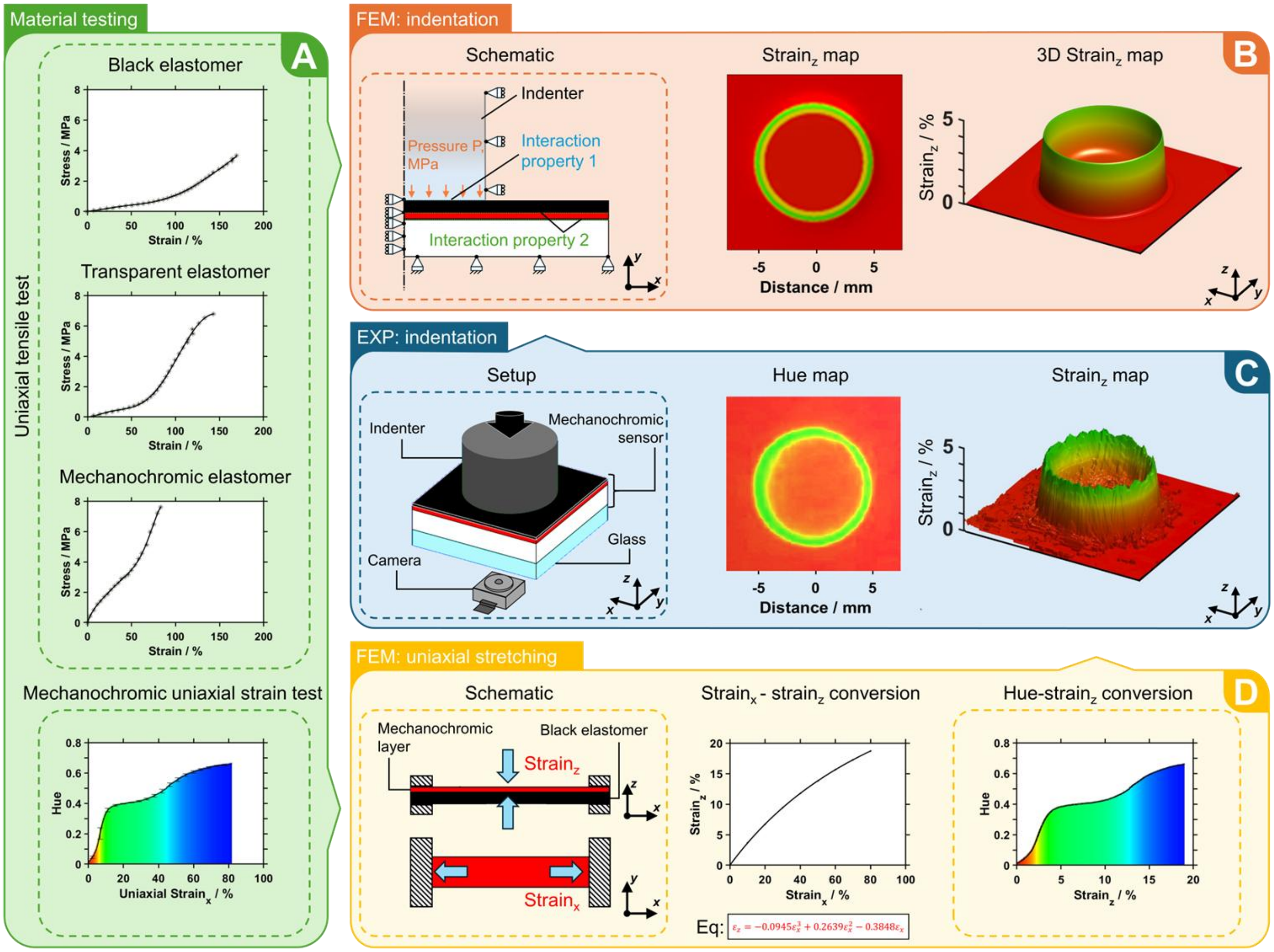

Fig. S6. FEM workflow. (**A**) Tests were performed to experimentally characterise both the uniaxial tensile stress-strain relationships of the constitutive elastomers (black elastomer, transparent elastomer, and mechanochromic elastomer) and the hue-uniaxial $Strain_x$ relationship of the mechanochromic elastomer. (**B**) FEM of the indentation tests was conducted to obtain simulated $Strain_z$ maps. (**C**) Experimental indentation tests were carried out, obtaining hue maps, which were then converted into $Strain_z$ maps, as explained by the next point. (**D**) A FEM-based uniaxial tension simulation was used to obtain a numerical relationship between $Strain_x$ and $Strain_z$, which was used in combination with the experimental hue-$Strain_x$ relationship, to estimate, from the hue maps measured during the indentations, the corresponding $Strain_z$ maps.

Tab. S1. Material parameters of Yeoh's hyperelastic model used for FEM.

Material	C10 (MPa)	C20 (MPa)	C30 (MPa)	Poisson's ratio
Black elastomer	0.4423	0.0151	0.0203	0.499995 (*)
Transparent elastomer	0.5582	0.1031	0.1063	0.499995 (*)
Mechanochromic elastomer	4.0283	-2.9956	3.1141	0.439000 (**)

(*) Assumption of nearly ideal incompressibility.

(**) B. H. Miller, H. Liu, M. Kolle, Scalable optical manufacture of dynamic structural colour in stretchable materials. *Nat. Mater.* **21**, 1014–1018 (2022) (*56*).

Tab. S2. FEM settings.

Parameter	Value
Analysis Type	Static, General
Rubber model type	Axisymmetric, Deformable
Indenter model type	Axisymmetric, Discrete rigid
Rubber element type	CAX4H: 4-Node bilinear axisymmetric quadrilateral, hybrid element
Indenter model type	RAX2: 2-Node linear axisymmetric rigid link
Global element size	0.045mm
Interaction property 1	Penalty (Friction Coeff: 1.5) & "Hard" Contact (separation disallowed)
Interaction property 2	Penalty (Friction Coeff: 0.5)
Resulting data	Nominal strain (y-axis), N22

Supplementary Movie S1: Surface topography mapping. The movie presents an example of the mechanochromic sensor performing a real-time tactile mapping of a one-penny coin's surface topography. The sensor was arranged in a robotic fingertip prototype, consisting of a transparent elastomer, embedding a camera surrounded by an LED ring. The fingertip was covered by a small circular patch of the mechanochromic bilayer, as detailed in the main text. The fingertip was made to slide over the surface, along two orthogonal diametral directions and a semi-circumferential path. For each case, the movie shows a collection of the following videos: an animation of the fingertip motion, a video recording of the fingertip motion, the raw video recording of the colour changes taken by the internal camera, and videos of the corresponding dynamic 2D and 3D hue maps.

Supplementary Movie S2: Contact morphology and strain mapping. The movie shows systematic investigations, through indentation tests, on the mechanochromic tactile sensor's ability to map the contact morphology and strain. The video array visualises dynamic $Strain_z$ maps, converted from experimental hue maps obtained during indentations with 3 mm and 10 mm-wide cylindrical indenters (schematically represented on the left side). The videos refer to different sensor configurations, varying in their layer thickness ratio h_1/h_2, as described in the main text. Each video ends when the maximum experimental pressure was reached.

Supplementary Movie S3: Contact pressure mapping. The movie shows an example of the use of the mechanochromic sensor in carrying out a real-time contact pressure mapping. As visible in the main video and in the schematic drawing, a subject was asked to write on the sensor's surface with a 3 mm-wide stylus the word 'HELLO'. The videos at the bottom show the instantaneous 3D hue map, a cumulative 3D hue map (obtained by combining sequential video frames to generate a composite image of the whole word), and pressure signals simultaneously recorded by the tactile sensor and load cell.
